# Livedoid Vasculopathy in a Young Woman With Elevated Factor VIII and Lipoprotein(a): A Case Report With Consideration of Future Pregnancy

**DOI:** 10.7759/cureus.115845

**Published:** 2026-09-06

**Authors:** Nicole Nudelman, Ann K Valencia, Alex Fernandez, Paige Johnson, Jeffrey Valencia Uribe, Brian G Nudelman

**Affiliations:** 1 Medicine, Kansas City University, Kansas City, USA; 2 Medicine, Florida International University Herbert Wertheim College of Medicine, Miami, USA; 3 Critical Care Medicine, University of South Florida, Pembroke Pines, USA; 4 Cardiology, Memorial Healthcare System, Pembroke Pines, USA

**Keywords:** anticoagulation in pregnancy, autoimmune, livedoid dermatitis, livedoid vasculitis, livedoid vasculopathy

## Abstract

Livedoid vasculopathy (LV) is a rare, chronic vascular disorder marked by painful, recurrent ulcerations that primarily affect the lower extremities. It is frequently associated with hypercoagulable states, although its pathophysiology remains incompletely understood. We present the case of a 25-year-old woman with a longstanding history of LV, elevated factor VIII, increased lipoprotein(a), recurrent kidney stones, and iron deficiency anemia who presented with worsening lower-extremity ulcerations. An extensive workup excluded other hypercoagulable and connective-tissue conditions, leaving elevated factor VIII and lipoprotein(a) as the most likely contributors to her disease. Although her symptoms initially resolved with treatment, they recurred over time, and management was further complicated by her desire for future pregnancy, which precluded the use of several standard anticoagulant therapies. Her regimen was ultimately centered on pentoxifylline and low-dose aspirin to control symptoms while minimizing risk to a future pregnancy. This case illustrates the importance of individualized treatment planning in LV, particularly when hypercoagulable risk factors intersect with reproductive goals.

## Introduction

Livedoid vasculopathy (LV) is a rare, chronic thrombotic disorder of the cutaneous microcirculation characterized by recurrent painful ulcerations that predominantly affect the lower extremities. It occurs most commonly in young to middle-aged women and has been associated with a variety of inherited and acquired prothrombotic conditions. Although its pathogenesis remains incompletely understood, microvascular thrombosis resulting from an imbalance between coagulation and fibrinolysis is thought to play a central role in its persistent and relapsing course [1].

We describe a 25-year-old woman with LV who was also found to have elevated lipoprotein(a) and factor VIII levels, abnormalities that may contribute to an increased thrombotic tendency but whose specific relationship to LV remains incompletely defined. These findings were clinically relevant because they provided additional context for evaluating her overall thrombotic risk and selecting an appropriate long-term treatment strategy. Management was further influenced by her desire for future pregnancy, which required consideration of the reproductive safety and suitability of available antithrombotic therapies. This report highlights the challenges of managing LV in the setting of concurrent prothrombotic risk factors and illustrates how reproductive planning may influence individualized treatment decisions.

## Case presentation

The patient is a 25-year-old woman with a past medical history LV, elevated factor VIII, increased lipoprotein(a), recurrent kidney stones, iron deficiency anemia, and extremity numbness. She presented to the clinic for follow-up after an emergency department visit for left upper extremity weakness and worsening lower-extremity ulcerations.

She first developed painful leg ulcerations at age 15, at which time she was followed closely by pediatric hematology/oncology and rheumatology. Workup at that time revealed an elevated factor VIII level (158%) and increased lipoprotein(a), while testing for connective tissue and rheumatologic disease was negative, and a comprehensive vitamin panel was within normal limits (Table 1). A biopsy of a lower-extremity ulcer, performed with pediatric dermatology, confirmed the diagnosis of LV. Treatment with doxycycline, pentoxifylline, aspirin, clindamycin, and naproxen led to complete resolution of her lesions.

**Table 1:** Laboratory findings and clinical significance Note: Reference ranges are representative and may vary by assay and performing laboratory. Abbreviations: ANA, antinuclear antibody; CRP, C-reactive protein; ESR, erythrocyte sedimentation rate; IFA, indirect immunofluorescence assay; INR, international normalized ratio; LV, livedoid vasculopathy; PT, prothrombin time; PTT, partial thromboplastin time; RBC, red blood cells; SGU, standard IgG anti-beta-2 glycoprotein I units; SMU, standard IgM anti-beta-2 glycoprotein I units

Test/Timepoint	Result	Reference Range	Interpretation	Clinical Significance
Age 15 evaluation				
Factor VIII activity	158%	50-150%	Elevated	Hypercoagulability risk factor; a recognized contributor to LV pathophysiology
Lipoprotein(a)	40 mg/dL	<30 mg/dL	Mild elevation	Associated with impaired fibrinolysis; implicated as a key driver of her LV
Beta-2 glycoprotein I antibodies	IgG <15 SGU	Negative (e.g., IgG <15 SGU; IgM <15 SMU)	Normal	Argues against antiphospholipid antibody syndrome
Lupus anticoagulant	Negative	Negative (assay-specific)	Normal	Argues against antiphospholipid antibody syndrome
ANA	1:40	Negative (commonly <1:80 by IFA; assay dependent)	Normal	Argues against systemic lupus erythematosus / connective tissue disease
Cryoglobulin	Negative	Negative	Normal	Argues against cryoglobulinemia
Cryofibrinogen	Negative	Negative	Normal	Argues against cryofibrinogenemia
ESR	10 mm/h	0–20 mm/h (representative)	Normal	No evidence of systemic inflammatory process
CRP	2.2 mg/L	<5 mg/L	Normal	No evidence of systemic inflammatory process
Rheumatoid factor	<15 IU/mL	<15 IU/mL	Normal	Argues against rheumatoid arthritis-associated vasculopathy
Comprehensive vitamin panel	Within normal limits	Analyte-specific	Normal	Nutritional deficiency excluded as contributor
Lower-extremity skin biopsy	Consistent with LV	N/A	Diagnostic	Confirmed diagnosis of livedoid vasculopathy
Age 21-22 (on pentoxifylline)				
Serum creatinine	1.35 mg/dL	0.59-1.04 mg/dL (adult female)	Abnormal	Led to discontinuation of pentoxifylline by adult rheumatology
Urinalysis	10-30 RBC/hpf	0-RBC/hpf (representative)	Abnormal	Contributed to discontinuation of pentoxifylline
Age 25 (current) thrombophilia workup				
Protein C activity	<150%	70-150%	Normal	Argues against protein C deficiency
Protein S activity	<150%	50-150% (female <50 years)	Normal	Argues against protein S deficiency
Prothrombin time (PT)	Within normal limits (exact time not available)	9.4-12.5 s; INR 0.9-1.1	Normal	No evidence of an extrinsic-pathway coagulopathy
Partial thromboplastin time (PTT)	Within normal limits (exact time not available)	25-37 s	Normal	No evidence of an intrinsic-pathway coagulopathy
Factor V Leiden mutation	Negative	Negative	Normal	Argues against factor V Leiden thrombophilia
Prothrombin gene mutation (G20210A)	Negative	Negative	Normal	Argues against prothrombin gene mutation thrombophilia

After a two-year symptom-free interval, she developed pain and swelling of the hands and feet with an itchy rash. Rheumatology restarted pentoxifylline (Trental) and referred her to physical therapy. After she transitioned to adult rheumatology care, pentoxifylline was discontinued because of rising creatinine and microscopic hematuria (Table 1). She remained asymptomatic for the following six years, managed with compression stockings alone.

At age 23, she presented to the emergency department with new bilateral lower-extremity ulcerations and swelling Figure 1. Rheumatology discharged her with doxycycline and prednisone and arranged outpatient follow-up, at which pentoxifylline was restarted, and topical mupirocin was added.

**Figure 1:**
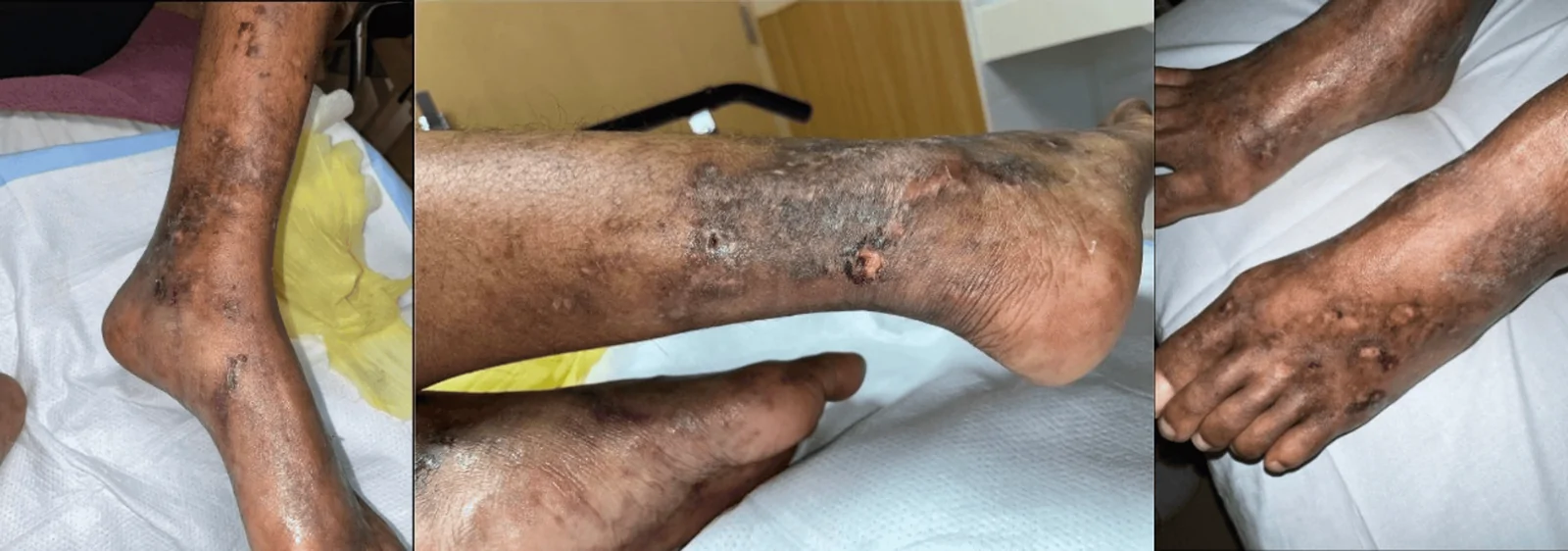
New lower extremity ulcerations seen in ED visit Lower extremities demonstrate retiform purpuric lesions with painful, irregular ulcerations and surrounding erythema, consistent with livedoid vasculopathy. Areas of healing show white, stellate atrophic scarring (atrophie blanche) with associated hyperpigmentation.

At her most recent clinic visit, she reported having stopped pentoxifylline after her prescription ran out. Examination revealed diffuse ulcers below both knees, including one painful, infected ulcer on the plantar aspect of the right foot. She was started on cephalexin and aspirin 81 mg twice daily and was referred to hematology/oncology. An extensive thrombophilia workup - including proteins C and S, prothrombin time (PT), partial thromboplastin time (PTT), factor V Leiden, and prothrombin gene testing - was negative (Table 1). Following consultation with specialists at a local university medical center, her regimen was finalized as pentoxifylline 400 mg three times daily plus aspirin 81 mg once daily. This regimen was created with consideration of all her associated comorbidities and reproductive considerations (Table 2).

**Table 2:** Comorbidities and reproductive considerations DOACs: direct oral anticoagulants

Comorbidity/Feature	Relevance to Case
Recurrent kidney stones	Noted in past medical history; not directly implicated in LV pathophysiology but part of overall clinical picture
Iron deficiency anemia	Noted in past medical history; monitored alongside other findings
Extremity numbness/left upper extremity weakness	Prompted the emergency department visit that led to the current clinic follow-up
Desire for future pregnancy	Excluded warfarin, DOACs, and danazol from the treatment plan; limited high-dose aspirin due to bleeding risk; shaped the final regimen of pentoxifylline plus low-dose aspirin

## Discussion

LV is rare, affecting an estimated one in 1,000,000 people annually, and typically presents with lower-extremity ulcerations in women between the ages of 15 and 50 [1]. Its pathophysiology involves increased thrombotic activity within the endothelium of dermal vessels together with reduced fibrinolytic activity, producing the disease's characteristic ulcerations [1]. Conditions frequently associated with LV include chronic venous hypertension, varicose veins, systemic lupus erythematosus, antiphospholipid antibody syndrome, factor V Leiden, prothrombin gene mutations, cryoglobulinemia, and myeloproliferative disorders [2]. Diagnosis is primarily clinical, though biopsy can support it by showing hyalinization and thickening of superficial dermal vessels with intraluminal fibrin deposits; direct immunofluorescence may additionally reveal complement and immunoglobulin deposition within the dermal vasculature [2].

Management of LV combines wound care, smoking cessation, and compression therapy, with intravenous immunoglobulin considered in select cases [2]. Pharmacologic options include pentoxifylline, aspirin, low molecular weight heparin, warfarin, direct oral anticoagulants, tissue plasminogen activator, danazol, doxycycline, and dapsone [2,3]. Peripheral vasodilators, such as nifedipine and cilostazol, have also shown benefit, likely through their vasodilatory effect [2]. LV follows a chronic course of remission and recurrence and has no definitive cure, and treatment efficacy data remain limited [2].

In this patient, an extensive workup excluded most conditions typically associated with LV, leaving elevated lipoprotein(a) and factor VIII as the most likely contributors to her disease. Lima et al. [4] described a young woman with LV and elevated lipoprotein(a) who achieved complete resolution of her ulcerations with pentoxifylline and aspirin. Criado et al. subsequently studied danazol in four LV patients with elevated lipoprotein(a) and found complete ulcer healing and pain reduction in all four, along with a roughly 70% reduction in lipoprotein(a) levels [3]. More recently, Huot et al. [5] documented successful treatment with rivaroxaban in a man with LV and elevated protein S, homocysteine, and factor VIII.

Because our patient desires future pregnancy, oral anticoagulation was not a viable option, and concerns about adherence similarly limited low molecular weight heparin. High-dose aspirin carried a meaningful risk of gastrointestinal bleeding, and danazol was contraindicated given its effects on pregnancy. For these reasons, treatment was continued with pentoxifylline and low-dose aspirin, with close follow-up planned with both our team and hematology/oncology to monitor her response [6].

## Conclusions

This case illustrates the chronic, relapsing nature of LV and the complexity of its management. Elevated lipoprotein(a) and factor VIII levels were identified in this patient and may have contributed to an underlying prothrombotic phenotype, although their specific role in the persistence of her disease remains uncertain. Management was further influenced by her desire for future pregnancy, which required careful consideration of the safety and suitability of available antithrombotic therapies.

Pentoxifylline and low-dose aspirin were selected as an individualized management strategy aimed at controlling symptoms while balancing thrombotic and reproductive considerations. Further research is needed to clarify the potential roles of lipoprotein(a) and factor VIII in LV and to better guide treatment in patients with similar clinical circumstances.
